# Response triggering by an acoustic stimulus increases with stimulus intensity and is best predicted by startle reflex activation

**DOI:** 10.1038/s41598-021-02825-8

**Published:** 2021-12-08

**Authors:** Dana Maslovat, Christin M. Sadler, Victoria Smith, Allison Bui, Anthony N. Carlsen

**Affiliations:** School of Human Kinetics, University of Ottawa, 125 University Private, Ottawa, ON Canada

**Keywords:** Neuroscience, Motor control, Neurophysiology

## Abstract

In a simple reaction time task, the presentation of a startling acoustic stimulus has been shown to trigger the prepared response at short latency, known as the StartReact effect. However, it is unclear under what conditions it can be assumed that the loud stimulus results in response triggering. The purpose of the present study was to examine how auditory stimulus intensity and preparation level affect the probability of involuntary response triggering and the incidence of activation in the startle reflex indicator of sternocleidomastoid (SCM). In two reaction time experiments, participants were presented with an irrelevant auditory stimulus of varying intensities at various time points prior to the visual go-signal. Responses were independently categorized as responding to either the auditory or visual stimulus and those with or without SCM activation (i.e., SCM+/−). Both the incidence of response triggering and proportion of SCM+ trials increased with stimulus intensity and presentation closer to the go-signal. Data also showed that participants reacted to the auditory stimulus at a much higher rate on trials where the auditory stimulus elicited SCM activity versus those that did not, and a logistic regression analysis confirmed that SCM activation is a reliable predictor of response triggering for all conditions.

## Introduction

In order to perform an intended movement, it is critical that the muscle commands be prepared, initiated and executed appropriately. While these processes clearly influence one another, there is substantial evidence that response preparation, initiation, and execution are separate and dissociable processes^1–3^. These processes can be independently examined through the use of a reaction time (RT) paradigm, in which a performer responds to an external go-signal by performing a required response as fast and accurately as possible. When the response is known prior to the go-signal (known as simple RT), the response latency is much shorter than when the go-signal cues the response (known as choice RT), which has been taken as evidence that preparation of the response occurred in advance of the go-signal^2,4^. This preprogramming indicates that preparatory processes can occur in isolation and that the prepared response can be held in readiness until the go-signal occurs^3^, with RT decreasing with increasing temporal predictability of the go-signal^5^. Furthermore, when the response has been prepared and held in advance, the response latency relative to the go-signal is largely indicative of initiation processes^1^, which have been shown to be accelerated when the go-signal is replaced with a loud acoustic stimulus capable of eliciting a reflexive startle response^6,7^. In these experiments the startling acoustic stimulus (SAS) results in a much shorter RT latency, yet the execution of the response is largely unaffected^3,8^; critically however, this so-called “StartReact” effect only occurs when the response is in a high state of preparation^9,10^.

Although it is clear that reacting to the louder go-signal results in faster initiation of a prepared response, the specific neural pathways involved in the StartReact effect remain a matter of debate^11^. Several studies have provided evidence that the prepared movement is stored and involuntarily released, primarily via subcortical/reticular structures that are also involved with the startle reflex pathway^6,12,13^. While other experiments have provided support for cortical involvement in the StartReact effect^14,15^, they still implicate an initiation circuitry involving subcortical structures that are different to the initiation pathways involved in reacting to a non-startling go-signal. In contrast, it has instead been argued that short latency responses following the loud go-signal involve normal voluntary initiation pathways and can be considered a special case of the stimulus intensity effect^16^, whereby the SAS acts as an accessory stimulus to increase neural activation above initiation threshold and thus involuntarily triggers the response at a reduced RT^17^.

It is perhaps this lack of agreement regarding the initiation pathways involved in the StartReact effect that has resulted in a variety of experimental methods, as well as differing interpretations of the results of trials involving the presentation of a SAS. For example, the loud acoustic stimulus has been delivered via a loudspeaker, headphones, and other creative methods such as discharging TMS over a metallic platform^6,7^, with stimulus intensities ranging from ~ 100^18,19^ to ~ 130 dB^6,20^. Similarly, there is a lack of consensus regarding trial inclusion/exclusion criteria, specifically whether there needs to be overt activation in the circuitry associated with the startle reflex. Those who consider the initiation pathway for the StartReact effect to involve subcortical structures argue that some indication of activation of these circuits is required, which is often confirmed by the presence of a startle reflex indicator^11,21^. This is typically achieved by ensuring there is observable short latency (< 120 ms) electromyographic activity in the sternocleidomastoid (SCM) muscle, which is thought to be a robust and reliable indicator of startle activity in reticular structures^22,23^. From this viewpoint, those trials which exhibit SCM activity (i.e., SCM + trials) are considered “true” startle trials and can be analyzed on their own or compared to those without SCM activity (i.e., SCM− trials). This SCM+/− comparison can be used to control for stimulus intensity effects associated with the louder go-signal and therefore used to confirm whether a greater reduction in RT is present on SCM+ versus SCM− trials, indicative of an effect due to activation of the startle reflex circuitry, rather than simply the intensity of the stimulus^20,24^. Consistent with this viewpoint, a recent meta-analysis has shown that for most responses SCM+ trials are performed at significantly shorter RT latency than SCM− trials^25^, which has been taken as evidence that faster initiation processes involving subcortical pathways occur when a startle reflex is observed.

Conversely, if the initiation pathway is thought to involve the same circuitry regardless of the intensity of go-signal used, confirmation of startle reflex activation is not a necessary requirement and thus SCM− trials are not discarded from the analysis^17,26^. From this perspective, the shorter latency RT seen on SCM+ trials is attributed to a greater level of preparation on these trials, which results in both shorter RTs and an increased likelihood of eliciting a startle reflex. In this manner, the relationship between shorter RT and SCM presence may not be a causal relationship indicative of different initiation pathways, and instead could be due to a common factor of increased preparation level^17^. Indeed, there is considerable overlap in the RT distributions between SCM+/− trials and reported mean RT differences between these trial types are within the range expected due to preparation differences^27^. While these different viewpoints have led to varying methodologies, the decision whether or not to include SCM- trials in the analysis of the StartReact effect is not a trivial one, as it has been shown that the inclusion versus exclusion of the SCM− trials can lead to very different experimental results and thus different conclusions regarding neural circuitry and response preparation processes^28^. Additionally, whether or not confirmation of SCM activation is required may affect the intensity of the SAS employed in a given study, as it is well known that a louder go-signal increases the likelihood of eliciting a startle reflex^21,29^.

Regardless of the specific pathways involved in the StartReact effect, most agree that the presentation of a loud acoustic stimulus can increase excitation in the response initiation circuitry, leading to involuntary response triggering at short latency^3,11,17,26^. However, whether the increased excitation caused by the SAS is sufficient to reach initiation threshold is likely dependent on the level of preparedness of the performer, as well as the intensity of the stimulus. For example, when presenting a very loud SAS (> 120 dB) in place of or at the same time as the non-startling go-signal, it is very likely that the high intensity SAS will provide the necessary input to involuntarily trigger the response, as the participant would be expected to be at a high level of preparation when the auditory stimulus is presented. However, response triggering may or may not occur when the stimulus intensity is lower, when the SAS is presented substantially earlier than the usual time of the go-signal (i.e., when preparation level is not maximized)^10,26,30^, or during dual-task performance^31,32^.

The primary purpose of the current study was to examine how stimulus intensity and preparation level affect the incidence of response triggering, SCM activation, and latency of the observed response. This was achieved in Experiment 1 by using a simple RT paradigm with a visual go-signal and presenting an irrelevant auditory stimulus of varying intensities (80 dB, 100 dB, 110 dB, 120 dB) either 300 ms or 1000 ms before the go-signal. The presentation of the auditory stimulus prior to the visual go-signal allowed for determination as to whether participants reacted to the auditory stimulus, as this would result in response onset occurring in advance of the visual go-signal. It was predicted that with increasing stimulus intensity, a corresponding increase in the proportion of SCM+ trials would be observed, as well as increased incidence of response triggering by the auditory stimulus. Similarly, it was predicted that participants would be at a higher level of preparatory activation closer to the usual time of the visual go-signal, which would be reflected in an increased likelihood of both startle-related activation in the SCM, as well as response triggering by the auditory stimulus. Lastly, it was predicted that for each auditory stimulus intensity level, the RT latency on SCM+ trials would be significantly shorter as compared to SCM- trials for those trials triggered by the auditory stimulus.

A secondary purpose was to evaluate startle-related SCM activation as a reliable and valid predictor of response triggering. To determine which experimental factors and measured variables were predictive of response triggering by the acoustic stimulus, logistic regression models were used to predict the binary outcome of whether or not a response would be triggered by the auditory stimulus. It was expected that SCM activation would be a robust predictor at all stimulus intensities and presentation times, as previous work has shown that response latencies for SCM+ trials are similar, irrespective of the intensity of the auditory stimulus^22,23^. Determining under what conditions an auditory stimulus reliably triggers a prepared response has important methodological implications for paradigms in which a SAS is used to probe response preparation. In addition, delineation of the relationship between startle reflex activity, response triggering, and response latency may provide additional information pertaining to the pathways involved in the StartReact effect.

Based on the results of Experiment 1, we conducted a second control experiment to examine why the observed incidence of response triggering for the 120 dB stimulus presented 300 ms before the go-signal was lower than expected. This condition involved the most intense stimulus presented at a time when participants should be at a very high preparation level, yet the prepared response was only triggered on 62% of trials. However, unlike typical StartReact paradigms, Experiment 1 presented participants with an auditory stimulus of varying intensity on half of the trials, which they were told was irrelevant to the task. Thus, a possible explanation for the lowered rate of response triggering is that the frequent auditory stimuli may have resulted in a “gating”/filtering of auditory input to focus on the visual go-signal. To test this hypothesis, we performed Experiment 2 in which auditory stimuli were presented on a smaller proportion of trials, similar to what would be performed in a more typical StartReact protocol. As the purpose of Experiment 2 was to concentrate on the examination of response triggering when participants were expected to be at a high level of preparation, only two high intensity auditory stimuli (114 dB, 120 dB) were presented on selected trials at 300 ms prior to the visual go-signal.

## Results

### Experiment 1: Startle indicators

**Figure 1:**
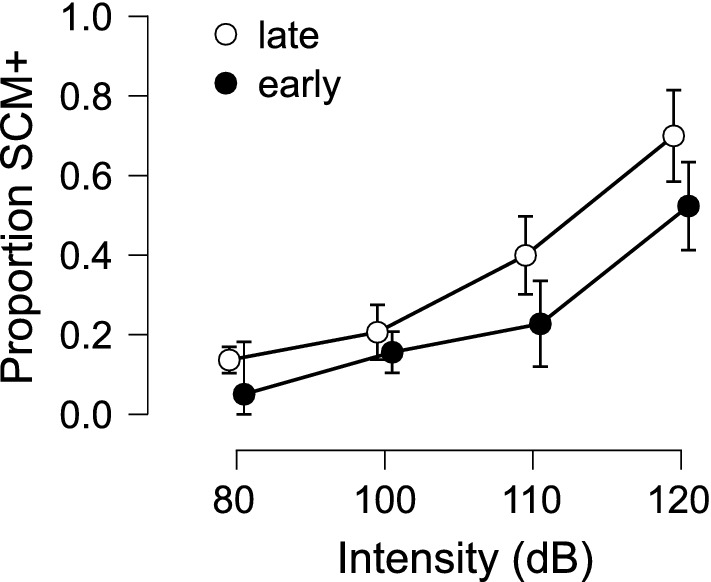
Mean proportion (error bars: 95% CI) of auditory stimulus trials where a short latency burst of EMG activity was observed in the sternocleidomastoid muscle (SCM) as a function of stimulus intensity (dB) and time of stimulus presentation (early: − 1000 ms, black circles; late: − 300 ms, white circles) with respect to the visual go-signal.

The proportion of auditory stimulus trials where a burst of EMG activity was observed in SCM is presented in Fig. 1. Repeated measures ANOVA confirmed a significant main effect of Presentation time, F(1,10) = 22.553, p < 0.001, η^2^_p_ = 0.693, and a significant main effect of Stimulus intensity, F(3,30) = 64.965, p < 0.001, η^2^_p_ = 0.867. The interaction between the factors was not significant, F(3,30) = 1.787, p = 0.171, η^2^_p_ = 0.152. Post hoc tests showed that the percentage of trials where a SCM response was elicited was significantly greater with each increase in intensity (all p-values < 0.027).

### Experiment 1: Incidence of response triggering

**Figure 2:**
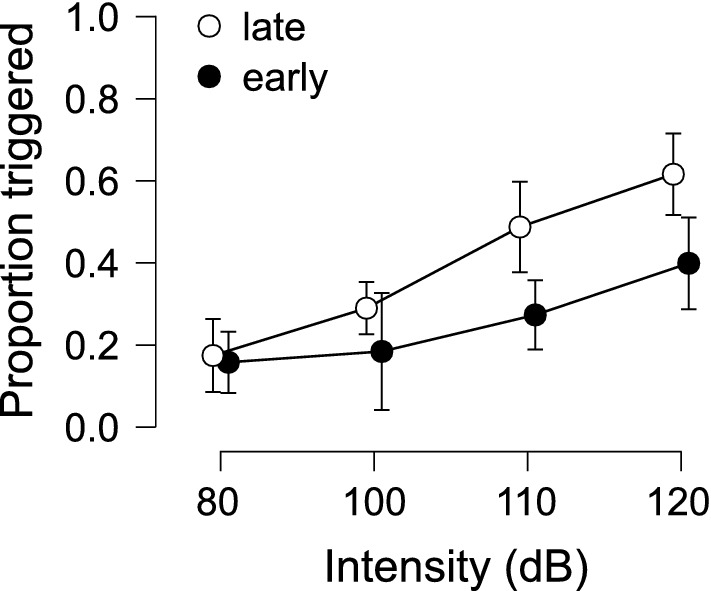
Mean proportion (error bars: 95% CI) of auditory stimulus trials where the prepared response was triggered within 300 ms of the stimulus as a function of stimulus intensity (dB) and time of stimulus presentation (early: − 1000 ms, black circles; late: − 300 ms, white circles) with respect to the visual go-signal.

The percentage of auditory stimulus trials where a response was triggered as a function of stimulus intensity and time of stimulus presentation is shown in Fig. 2. Repeated measures ANOVA confirmed a significant main effect of Presentation time, F(1,10) = 35.814, p < 0.001, η^2^_p_ = 0.782, indicating that a later presentation time led to a larger proportion of trials where the response was triggered by the auditory stimulus. There was also a significant main effect of Stimulus intensity, F(3,30) = 18.628, p < 0.001, η^2^_p_ = 0.651, where each increasing intensity above 100 dB led to an increased proportion of trials that were triggered by the auditory stimulus (the difference between 80 and 100 dB was not significant, p = 0.076). The interaction between the factors was not significant, F(3,30) = 1.884, p = 0.154, η^2^_p_ = 0.159.

**Figure 3:**
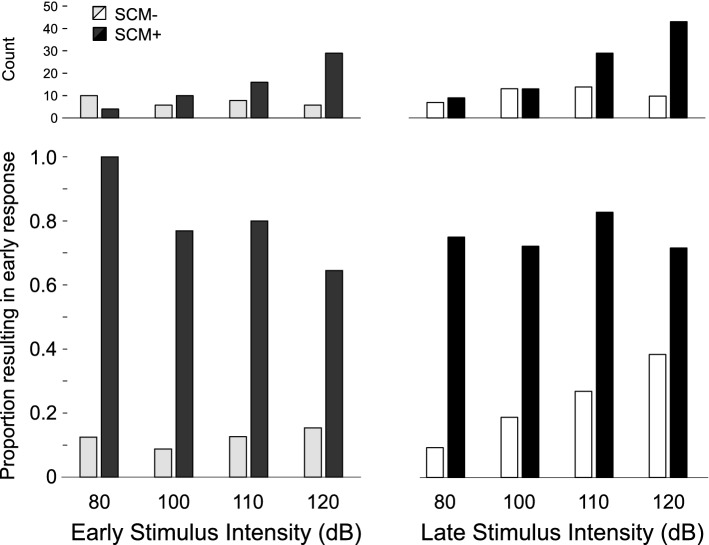
Proportion (bottom panels) and number (top panels) of trials where sternocleidomastoid (SCM) EMG activity was (SCM+, black/dark grey) or was not (SCM−, white/light grey) observed that resulted in triggering of the prepared response, as a function of stimulus intensity (dB) and time of stimulus presentation (early: − 1000 ms; late: − 300 ms). As not all participants showed SCM+ or SCM− for all conditions, the proportional data represents a grand mean across all participants.

While the previous analysis compared the proportion of all trials where triggering was observed as a function of intensity and time, it is also informative to examine the proportion of SCM+ and SCM− trials that led to response triggering. However, because all participants did not exhibit SCM+ and SCM− trials at all intensities, this variable could not be analyzed using repeated measures ANOVA, as there were many missing cells. Nevertheless, a descriptive analysis and plot of composite percentage data is provided below (Fig. 3, bottom panels). In general, it can be seen that for both stimulus presentation times and for intensities of 100–120 dB, when EMG activity was observed in SCM (i.e., SCM+), an early response was triggered on 79% of trials (minimum 64%), whereas for SCM− trials a response was triggered on less than 40% of trials (18% on average). Also shown in Fig. 3 are the total number of trials triggered at each intensity level and presentation time, separated by the presence/absence of SCM activation (top panels).

### Experiment 1: Premotor RT

**Figure 4:**
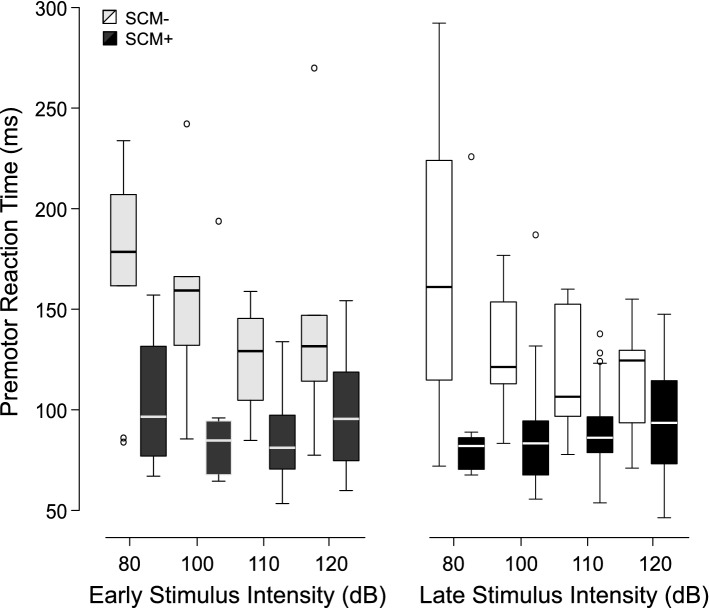
Boxplots of premotor reaction time as a function of stimulus intensity (dB), time of stimulus presentation (early: − 1000 ms; late: − 300 ms), and whether sternocleidomastoid (SCM) EMG activity was (SCM+, black/dark grey) or was not (SCM−, white/light grey) observed. Box boundaries represent first and third quartiles, the horizontal line indicates the median, and error bars extend to the farthest data point within 1.5 times the interquartile range from the boundary. Small circles represent outlier data points.

Premotor RT for trials where the auditory stimulus resulted in early response triggering is shown in Fig. 4. Linear Mixed Effects (LME) analysis confirmed a main effect of Presentation time, F(1,202.365) = 4.014, p = 0.046, where RT for the later stimulus time (118 ms, 95% CI [105, 131]) was shorter than for the early stimulus time (128 ms, 95% CI [115, 142]). There was also a main effect of SCM presence, F(1,9.885) = 26.934, p < 0.001, where RT for trials with SCM activity (103 ms, 95% CI [91, 116]) was shorter than for trials with no SCM activity (143 ms, 95% CI [125, 161]). There was also a main effect of Stimulus intensity, F(3,192.327) = 8.013, p < 0.001; however, this effect was superseded by an interaction between SCM presence and Intensity, F(3,174.054) = 3.255, p = 0.023. Post-hoc tests showed that for SCM+ trials there was no difference in RT for any of the intensities (all p-values > 0.842). For SCM− trials, RT appeared to decrease with increasing intensity (see Fig. 4); however, RT was only significantly longer following the 80 dB stimulus, as compared to all other stimuli (p-values < 0.011). One reason for the lack of difference between the intensities may be the relatively small number of trials where response triggering occurred in the absence of SCM (< 14 total trials per condition; see Fig. 3).

### Experiment 1: Predictors of response triggering

**Figure 5:**
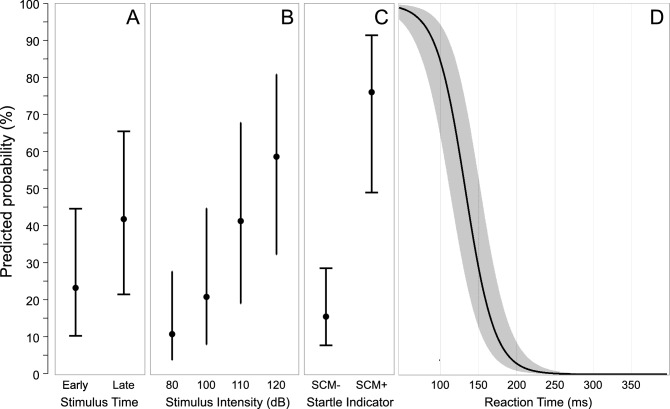
Predicted probability (error bars: 95% CI) of observing the triggering of the prepared response for the individual predictors from the binomial generalized linear mixed model analysis. (**A**) shows probability based on stimulus presentation time (early: − 1000 ms; late: − 300 ms). (**B**) shows probability based on auditory stimulus intensity (dB). (**C**) shows probability based on whether sternocleidomastoid (SCM) EMG activity was (SCM+) or was not (SCM−) observed. (**D**) shows probability based on reaction time (ms).

Logistic regression models using individual predictors of response triggering are provided in Fig. 5 (logits transformed to probability, 95% CI error bars). The model including only stimulus presentation time (Fig. 5A; early vs late) confirmed that time was a significant predictor of response triggering (β = 0.866, p < 0.001, AIC = 672.6), indicating that when the stimulus was presented later, the odds of triggering a response were 2.38 times higher than when presented early. For intensity alone (Fig. 5B), all intensities significantly contributed to the model (all p values < 0.027, AIC = 618.0), and pairwise comparisons indicated that while 80 dB and 100 dB did not differ in their predictive ability (p = 0.120), 110 dB had a significantly higher predictive ability than 80 dB (p < 0.001) and 100 dB (p = 0.005). In addition, 120 dB had a significantly higher predictive ability than 80 dB (p < 0.001), 100 dB (p < 0.001) and 110 dB (p = 0.034). The presence of EMG activity in SCM was also a significant predictor of response triggering (β = 2.838, p < 0.001, AIC = 568.5) indicating that when SCM activity was present, the odds of triggering a response were 17.1 times higher than if no SCM activity was observed (Fig. 5C). Finally, RT (adjusted to the initiating stimulus) was found to be a significant predictor of response triggering (β = − 0.052, p < 0.001, AIC = 431.7) with a significantly lower likelihood of response triggering seen with longer latency RTs (Fig. 5D).

**Figure 6:**
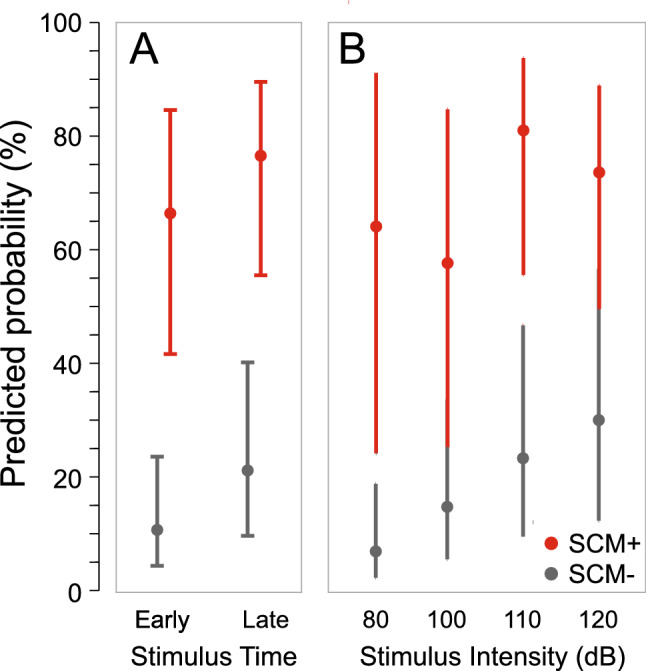
Predicted probability (error bars: 95% CI) of observing the triggering of the prepared response based on whether sternocleidomastoid (SCM) EMG activity was (SCM+, red) or was not (SCM−, grey) observed as a function of stimulus presentation time (early: − 1000 ms; late: − 300 ms; **A**), or stimulus intensity (dB; **B**).

**Figure 7:**
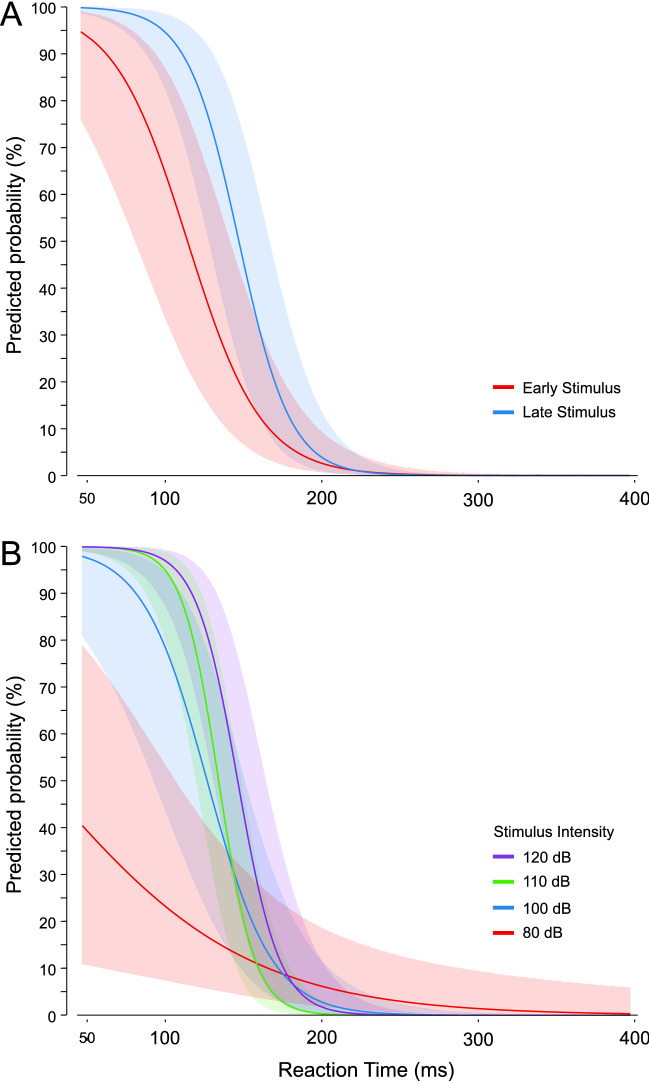
Predicted probability (error bars: 95% CI) of observing the triggering of the prepared response based on premotor reaction time (ms) as a function of stimulus presentation time (**A**; red: − 1000 ms; blue: − 300 ms), or stimulus intensity (**B**; red: 80 dB; blue: 100 dB; green: 110 dB; purple: 120 dB).

The models involving interactions between individual independent and dependent variables are provided in Figs. 6 and 7 (logits transformed to probability, 95% CI error bars). For the model involving an interaction between stimulus presentation time and SCM presence (Fig. 6A; AIC = 560.5), both factors contributed significantly to the model (time β = 0.801, p = 0.005; SCM β = 2.791, p < 0.001); however, the interaction between the factors was not a significant contributor (β = 0.302, p = 0.519). Similarly, for the model with an interaction between stimulus intensity and SCM presence (Fig. 6B; AIC = 557.0), stimulus intensities greater than 100 dB significantly contributed to the model (β values > 1.392, p values < 0.002) and SCM presence also significantly contributed (β = 3.153, p < 0.001); however, the interaction terms of the model did not provide a significant contribution (β values < 1.293, p-values > 0.145).

In the model including an interaction between stimulus presentation time and RT (Fig. 7A; AIC = 404.4), both factors significantly contributed to the predictive ability (time β = 4.085, p < 0.001; RT β = − 0.042, p < 0.001), and there was a significant interaction effect (β = − 0.018, p = 0.049). Furthermore, in the model including an interaction between stimulus intensity and RT (Fig. 7B; AIC = 383.7), all intensities significantly contributed to the model (β values > 5.757, p values < 0.002), RT significantly contributed (β = − 0.015, p = 0.009), and the interaction terms involving RT and intensity were all significant (β values < − 0.032, p-values < 0.005).

**Figure 8:**
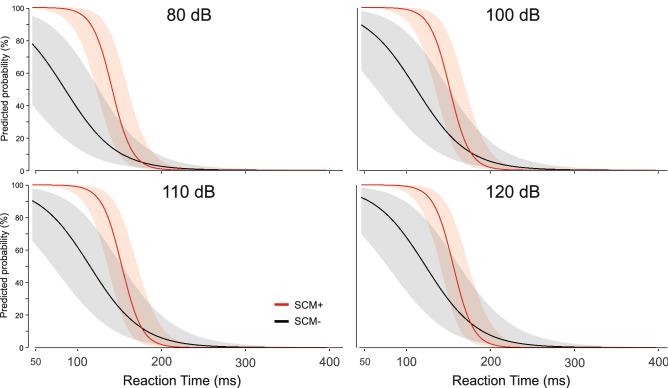
Predicted probability (error bars: 95% CI) of observing the triggering of the prepared response based on whether sternocleidomastoid (SCM) EMG activity was (SCM+, red) or was not (SCM−, grey) observed and premotor reaction time, separated by stimulus intensity (dB; separate panels). Note that models also include stimulus presentation time (early, late) as a factor but are collapsed across this factor for ease of presentation.

The model that provided the best fit (AICc = 361.0; null model 689.5) included SCM presence and RT as interacting factors with intensity and stimulus presentation time as additive factors (AIC = 360.7). The fixed effects are provided in Table 1 (note the intercept for categorical variables includes 80 dB/SCM−/Time-early). All factors significantly contributed to the model (except for the 100 dB intensity, p = 0.084). The full model is presented in Fig. 8 (collapsed across stimulus presentation time for ease of visualization).

**Table 1:** Fixed effects estimates (log odds) from the binomial generalized linear mixed model including reaction time (RT), whether sternocleidomastoid (SCM) EMG activity was (SCM+) or was not (SCM−) observed, stimulus intensity (in dB), and time of auditory stimulus presentation (early, late).

	Estimate	Std. error	z-value	P (> |z|)	Significant
(Intercept)	2.377	1.057	2.248	0.025	*
RT	− 0.033	0.005	− 6.439	< 0.001	*
SCM+	8.843	2.112	4.186	< 0.001	*
100 dB	0.796	0.4603	1.728	0.084	
110 dB	0.939	0.465	2.019	0.044	*
120 dB	1.345	0.515	2.617	0.009	*
Time-late	1.215	0.308	3.944	< 0.001	*
RT:SCM+	− 0.051	0.014	− 3.622	< 0.001	*

Note that the intercept includes the categories of SCM−, 80 dB, and early stimulus presentation time. The estimate of the change in log odds (SE) for an increment in each variable is provided along with the z-value of the logit and the p-value.

**Figure 9:**
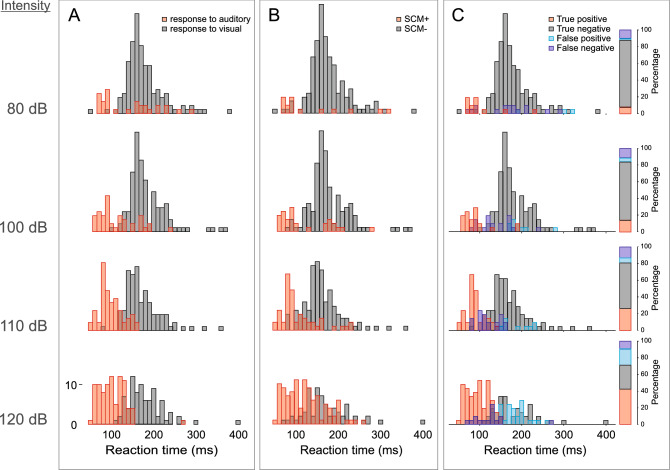
Histograms of premotor reaction time (RT) in 10 ms bins. (**A**) shows RT histograms as a function of stimulus intensity (arranged vertically) separated based on whether a response was made within 300 ms following the auditory stimulus (triggered, red) or made following the visual stimulus (not triggered, grey). (**B**) shows RT histograms as a function of stimulus intensity separated based on whether sternocleidomastoid (SCM) EMG activity was (SCM+, red) or was not (SCM−, grey) observed. (**C**) shows RT histograms as a function of whether the presence of SCM activity correctly predicted triggering (red: true positive; grey: true negative, blue: false positive; purple: false negative). The bar on the right of (**C**) also shows the percentage of RTs that fall into each of the aforementioned categories.

To summarize the logistic regression results, both SCM presence and RT latency were significant predictors of response triggering by the auditory stimulus. Whereas the predictive ability provided by presence of SCM activity did not change with either stimulus presentation time or stimulus intensity, the predictive ability of RT latency (particularly for positive responses) was significantly impacted at lower stimulus intensities and early presentation times. Nevertheless, the model that best predicted response triggering was one that considered both SCM presence and RT. To provide further detail on the relationship between SCM activation, response latency and triggering incidence, Fig. 9 shows the RT histograms for the auditory condition. Panel A shows RT separated based on SCM presence/absence irrespective of whether the response was triggered. Panel B shows RT separated based on whether the response was triggered by the auditory stimulus irrespective of SCM presence (note that these histograms reflect mostly the same bins of RT). Finally, panel C shows RT histograms categorized as true positives (SCM+ trial correctly identified as triggered), false positives (SCM+ trial incorrectly identified as triggered), true negatives (SCM- trial correctly identified as not triggered) and false negatives (SCM- trial incorrectly identified as not triggered). These results indicate that while true positives increase and true negatives decrease as stimulus intensity increases, the vast majority (> 70%) of trials are correctly identified by the presence/absence of SCM activation.

### Experiment 2: Startle indicators

The proportion of auditory stimulus trials where a burst of EMG activity was observed in SCM was significantly larger for the 120 dB stimulus (mean = 0.828, SD = 0.184) than for the 114 dB stimulus (mean = 0.708, SD = 0.203), t(9) = 4.594, p = 0.001, d = 1.453.

### Experiment 2: Incidence of response triggering

The proportion of auditory stimulus trials that led to early triggering of the response was not different between the 120 dB stimulus (mean = 0.842, SD = 0.215) and the 114 dB stimulus (mean = 0.830, SD = 0.217), t(9) = 0.497, p = 0.631, d = 0.157.

**Figure 10:**
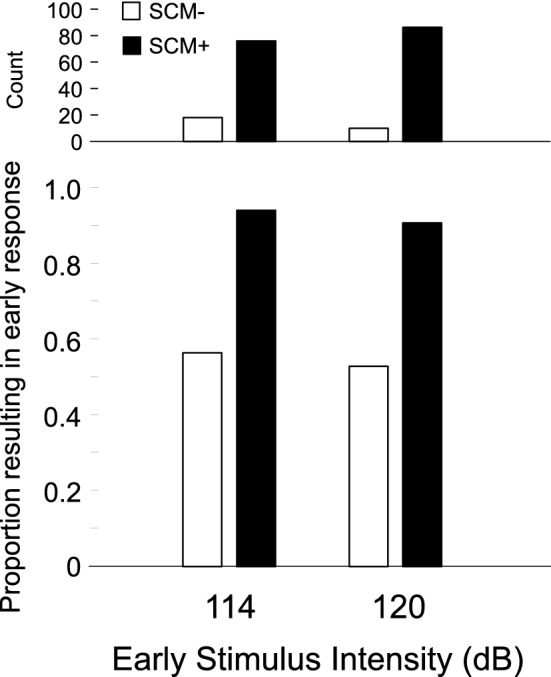
Proportion (bottom panel) and number (top panel) of trials where sternocleidomastoid (SCM) EMG activity was (SCM+, white) or was not (SCM−, black) observed that resulted in triggering of the prepared response, as a function of stimulus intensity (dB). As not all participants showed SCM+ or SCM− for all conditions, the proportional data represents a grand mean across all participants.

Similar to Experiment 1, not all participants exhibited SCM+ and SCM− trials at both intensities, and therefore the proportion of SCM+ and SCM− trials that led to response triggering could not be analyzed using repeated measures ANOVA. As such only a descriptive analysis and plot of composite percentage data is provided below (Fig. 10). In general, at both intensities when SCM activity was observed the response was triggered in > 90% of trials; however, when no SCM activity was observed, triggering by each stimulus only occurred on 56% or less of trials.

### Experiment 2: Premotor RT

**Figure 11:**
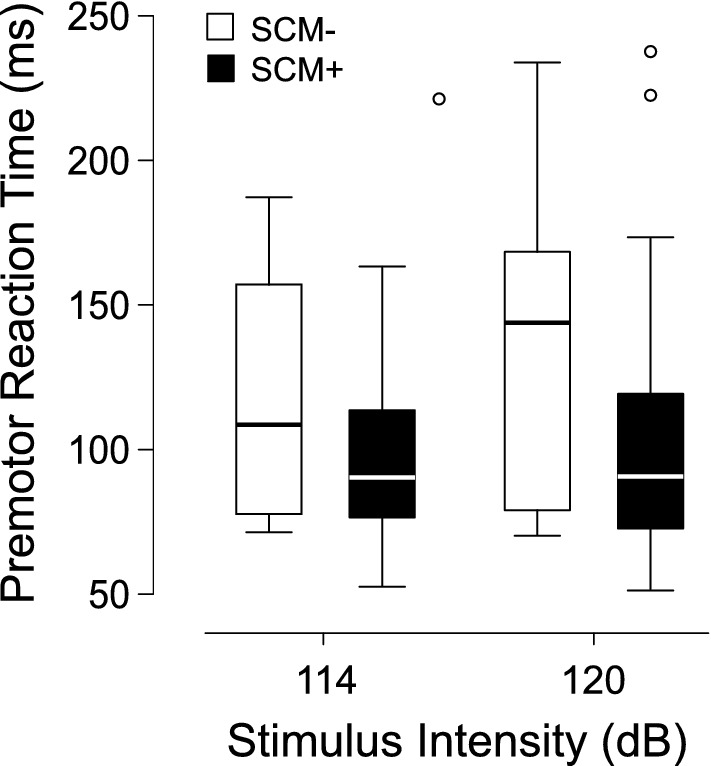
Boxplots of premotor reaction time as a function of stimulus intensity (dB), and whether sternocleidomastoid (SCM) EMG activity was (SCM+, black) or was not (SCM−, white) observed. Box boundaries represent first and third quartiles, the horizontal line indicates the median, and error bars extend to the farthest data point within 1.5 times the interquartile range from the boundary. Small circles represent outlier data points.

Premotor RT for trials where the auditory stimulus resulted in early response triggering is shown in Fig. 11. LME analysis confirmed a main effect of SCM presence, F(1,182.9) = 5.981, p = 0.015, where RT for trials with SCM activity (102 ms, 95% CI [84, 120]) was shorter than for trials with no SCM activity (118 ms, 95% CI [98, 138]). The main effect of Stimulus intensity was not significant, F(1,177.6) = 0.401, p = 0.527, and there was no significant interaction between the factors, F(1,177.4) = 0.555, p = 0.457.

### Experiment 2: Predictor of response triggering

**Figure 12:**
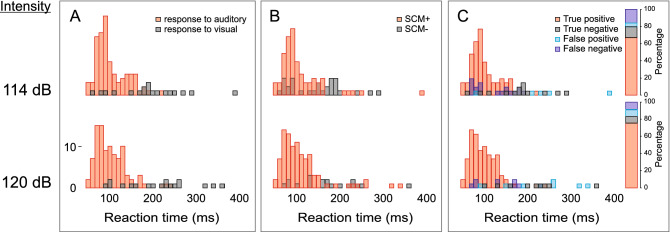
Histograms of premotor reaction time (RT) in 10 ms bins. (**A**) shows RT histograms as a function of stimulus intensity (arranged vertically) separated based on whether a response was made within 300 ms following the auditory stimulus (triggered, red) or made following the visual stimulus (not triggered, grey). (**B**) shows RT histograms as a function of stimulus intensity separated based on whether sternocleidomastoid (SCM) EMG activity was (SCM+, red) or was not (SCM−, grey) observed. (**C**) shows RT histograms as a function of whether the presence of SCM activity correctly predicted triggering (red: true positive; grey: true negative, blue: false positive; purple: false negative). The bar on the right of (**C**) also shows the percentage of RTs that fall into each of the aforementioned categories.

Similar to Experiment 1, the predictive ability of SCM activation was assessed by categorizing all auditory trials as true positives (SCM+ trial correctly identified as triggered), false positives (SCM+ trial incorrectly identified as triggered), true negatives (SCM− trial correctly identified as not triggered) and false negatives (SCM− trial incorrectly identified as not triggered). These results are shown in Fig. 12C and are consistent with data from Experiment 1 whereby true positives increase and true negatives decrease as stimulus intensity increases, and the vast majority of trials (≥ 80%) are correctly identified by the presence/absence of SCM activation.

## Discussion

The use of a loud acoustic stimulus to trigger a prepared movement has become an increasingly common methodology to examine response preparation, initiation, and execution. However, the protocols used in such experiments vary widely, including the intensity of the auditory stimulus and whether activation in a startle reflex indicator such as the SCM is assessed. This has led to not only a proliferation of different experimental methods, but also contrasting interpretations of results in such experiments, including which central nervous system pathways are involved in the triggering of the observed response. The purpose of the present study was twofold: (1) to examine how stimulus intensity and preparation level affect the triggering of a prepared movement, incidence of startle reflex activation in the SCM, and latency of the observed response; and (2) to determine if the presence of startle reflex-related SCM activation is a reliable and valid predictor of response triggering. The presentation of an irrelevant auditory stimulus of varying intensities either 1000 ms or 300 ms prior to the visual go-signal resulted in participants either responding to the auditory tone, which was deemed response triggering, or responding to the visual go-signal, which was deemed voluntary response initiation. In addition, and independent of whether the response was triggered by the auditory stimulus, trials were considered to either be SCM+ or SCM−, depending on whether there was short latency activation observed in the startle reflex indicator muscle. As predicted, the data clearly indicated that both SCM+ incidence and response triggering increased with stimulus intensity and preparation level (Figs. 1 and 2), and that these SCM+ trials were performed at shorter latency than the SCM− trials (Figs. 4 and 11). Also in line with the predictions, the logistic regression analysis provided strong evidence that SCM activation is a reliable predictor of response triggering, regardless of the intensity or presentation time of the auditory stimulus (Figs. 5 and 6). Overall, these novel results provide strong indication that when using a loud auditory stimulus to trigger a response under varying experimental and task parameters, trials can be deemed to have a high likelihood of having been triggered by the stimulus if they show activation in the SCM. Furthermore, the number of these trials can be maximized using a more intense stimulus presented when participants are at a high preparation level.

It has been well documented that a greater proportion of SCM+ trials is observed with increasing stimulus intensity and if the auditory stimulus is presented when participants are in a heightened state of preparation^21,29,30^. By presenting different stimulus intensities at two time intervals prior to the go-signal, the current study allowed for a more nuanced investigation into the characteristics of trials that were triggered by the auditory tone. In the various analyses used here, the data clearly indicate that the presence/absence of SCM activation is a robust indicator of whether or not a response was triggered by the loud acoustic stimulus irrespective of the level of preparation or intensity of the stimulus. In Experiment 1, the wrist extension movement was triggered following the auditory stimulus much more often on SCM+ trials (79%) than on SCM− trials (18%) for all intensities above 80 dB (Fig. 3, bottom panel). Although the incidence of response triggering was similar for SCM+ trials at the stimulus intensities of 100 dB, 110 dB, and 120 dB whether it was presented at 1000 ms or 300 ms prior to the visual go-signal, it is important to note that the absolute number of trials that resulted in response triggering was highest for the most intense stimulus presented closest to the go-signal (Fig. 3, top panel). Similar results were obtained in Experiment 2 when the auditory stimulus was either 114 dB or 120 dB and was always presented 300 ms prior to the visual go-signal, whereby 92% of SCM+ trials resulted in response triggering, as compared to 55% of SCM− trials (Fig. 10). Of note in Experiment 2 is that even though 55% of SCM− trials resulted in triggering, only a small number of trials were classified as SCM− (28 trials, or 12% of auditory stimulus trials). In addition, and consistent with previous results, early responses that were accompanied by a burst of EMG activity in SCM were significantly shorter than those without SCM activity (Figs. 4 and 11) and did not appear to be differentially impacted by the intensity of the stimulus.

These results provide confirmation of the efficacy of using SCM+ trials as an indicator of response triggering, although the data also indicate that this is not an “all or none” phenomenon. The RT histograms (Figs. 9 and 12) show that SCM+ trials did not result in 100% response triggering, nor did SCM− trials result in 0% response triggering; however, the proportion of false positive (SCM+ without response triggering) and false negative (response triggering in the absence of SCM activation) classifications were relatively small. Conversely, if all loud stimulus (> 100 dB) trials were assumed to be triggered, ignoring the presence/absence of SCM activation, it is clear this combination would result in a larger mix of trials in which triggering did and did not occur, leading to potentially erroneous conclusions. Furthermore, the SCM+/− delineation becomes even more important if the stimulus intensity is not maximized (i.e., ≥ 120 dB) or preparation levels are lowered, as these conditions result in a greater proportion of SCM− trials, which in turn have a much smaller incidence of response triggering by the auditory stimulus. Indeed, previous research has shown that when a 114 dB stimulus is presented prior to the go-signal, the requirement for SCM activation on startle trials results in substantially different conclusions than when all auditory trials are assumed to trigger the prepared response^28^.

Binary logistic regression models were also used to determine which variables were predictive of whether the response would be triggered by the auditory stimulus. Although stimulus intensity alone was a significant predictor of response triggering, it was only predictive at greater than 50% for the 120 dB stimulus (Fig. 5B), further supporting the notion that it is inappropriate to simply assume that loud stimuli will lead to response triggering. Similarly, a later stimulus presentation time, considered here to be indicative of greater preparatory levels, was a significant predictor of response triggering, but when grouped with other factors was ineffective as a predictor on its own (Fig. 5A). On the other hand, the presence of SCM activation showed a strong predictive ability with an estimated 76% probability of response triggering when SCM activation was noted, more than a 17 times greater likelihood than when no SCM activation was observed (Fig. 5C). Finally, RT alone also showed a significant predictive ability, particularly at the shortest RTs; however, to match the performance of SCM as a predictor, only RTs < 110 ms could be considered (Fig. 5D). Looking at Fig. 9, it is clear that this only represents a very small minority of trials for intensities of 110 dB or below. Although RT latency alone and SCM presence alone were significant predictors of response triggering, logistic regression models including additional predictors indicated that the predictive ability of the presence of SCM activity did not change with stimulus intensity or presentation time (Fig. 6), whereas the predictive ability of RT latency was weakened at lower stimulus intensities and the earlier presentation time (Fig. 7). Lastly, when SCM+/− trials were categorized as true positives, true negatives, false positives, and false negatives, the data showed that when using SCM activation as an indicator of response triggering, the vast majority of trials were correctly identified (true positives and true negatives), regardless of stimulus intensity or presentation time (> 70%, Figs. 9 and 12).

Although the recommendation for including only trials with distinct SCM activation has been previously made in the startle literature^11,21^, this criterion is not universally accepted and some studies have failed to find a RT difference between SCM+/− trials^33–35^. More recently, it has been suggested that rather than using SCM activity to examine response triggering, trials can instead be classified using a cumulative distribution function (CDF) of the response latency^36^. In this approach, participant RT data distributions are broken into quantiles and those responses in the 0–45% quantiles (fast onset) are considered triggered and those responses in the 55–100% quantiles (slow onset) are considered not triggered. The authors favored this approach due to what they consider to be the unreliability of SCM activation as it relates to response triggering, as well as the ability to use trials from all participants irrespective of whether they exhibit a classic startle reflex, thus maximizing the return from the collected data whist minimizing participant burden. However, the current data highlight potential problems with this CDF approach. First, although logistic regression analysis indicated that while RT can be a good predictor of response triggering, its predictive ability degrades with less intense stimuli and lowered preparation levels (Fig. 7). On the other hand, logistic regression indicated that SCM activation is also a robust predictor of response triggering, and this did not interact with stimulus intensities or presentation time (Fig. 6). Similar to how SCM+/− is not an “all or none” phenomenon, a particular RT latency quantile is also not always a reliable predictor of response triggering. The RT histograms shown in Fig. 9 clearly show that responses that are triggered by the auditory stimulus are more likely to result in earlier RTs, and are also more likely to be accompanied by SCM activation, but both approaches would result in false negatives and false positives. Secondly, the CDF approach used a 0–45% and 55–100% cutoff for fast and slow responses, yet the proportion of triggered trials is clearly dependent upon the stimulus intensity and presentation time (Figs. 7 and 9). By presenting the auditory stimulus in advance of the visual go-signal, the current study allowed for clear delineation of response triggering, and reported triggering incidences ranged from less than 20% for the early 100 dB in Experiment 1 to more than 90% for the 120 dB stimulus in Experiment 2. This means that using CDF analyses to delineate triggered from non-triggered trials would require constant adjustment of the quantiles in a task-dependent manner, indicating that its usefulness as a generalized predictor appears to be limited.

The current data provide strong confirmation that SCM activation is a robust predictor of response triggering, irrespective of the testing conditions. In addition, the inclusion of only SCM+ trials may be an important consideration depending on the pathways and processes assumed to be involved in this initiation process. Using SCM activation as a marker for response triggering also provides confirmation that the reflexive startle pathway has been engaged, a necessity if the StartReact effect is thought to depend upon activation in reticulospinal structures. If the response triggering occurs due to increased involvement of subcortical structures during the StartReact effect^6,37^, then an observable burst of short-latency SCM activity may indicate that these reticulospinal pathways are activated to the threshold needed to engage this faster initiation pathway. Alternatively, if the response triggering pathway is the same for all trials and the auditory stimulus simply acts as additional and accessory activation^17,26^, then SCM+ trials may represent those trials in which preparation levels are higher, and thus it is more likely the additional input caused by the auditory stimulus allows the system to reach the initiation threshold faster. It is important to note that although these viewpoints differ on the specific pathways involved in response triggering, both perspectives would predict that SCM+ trials would result in a greater likelihood of the auditory stimulus automatically initiating the prepared response (and shorter RT latency on SCM+ versus SCM− trials, as previously shown^25^, and replicated in both current experiments: Figs. 4 and 11).

The data from Experiment 1 were consistent with the predicted results, yet there was a relatively low incidence of response triggering (62%) when the most intense auditory stimulus (120 dB) was presented 300 ms prior to the go-signal. Furthermore, this was accompanied by a higher proportion of false positive responses than seen in other conditions (Figs. 9 and 12). It was hypothesized that this result may have been due to the frequent (50%) presentation of irrelevant auditory stimuli, which may have resulted in either the “gating”/filtering of auditory input, or increased motor inhibition related to an attempt to not respond to the irrelevant stimulus. Indeed, if higher levels of response inhibition were present alongside a highly prepared response, then a higher proportion of trials where SCM activation was observed but no response is triggered would be predicted. Therefore, in Experiment 2 the proportion of trials with an auditory stimulus was reduced to 20%, with a 114 dB or 120 dB SAS presented 300 ms prior to the visual go-signal. As expected and in line with the hypothesis, the presentation of a less frequent and more intense auditory stimulus resulted in a high percentage of SCM+ trials (71% for 114 dB, 83% for 120 dB), as well as a much higher incidence of response triggering on SCM+ trials (83% for 114 dB, 84% for 120 dB). These results indicate that when an infrequent, high intensity auditory stimulus is presented when participants are expected to be at a high level of preparation, the vast majority of SCM+ trials are due to response triggering by the SAS. Additionally, even when an infrequent and high intensity SAS was presented close to the expected go-signal, if SCM activation was absent response triggering only occurred on approximately half of the trials (56% for 114 dB, 52% for 120 dB), although this only represents a small minority of total trials.

Although the current data delineate the relationship between the stimulus characteristics and involuntary response triggering, it should be noted that the results are based on relatively small sample sizes (Experiment 1, n = 11; Experiment 2, n = 10). However, the large effect sizes associated with stimulus intensity and time of stimulus presentation suggests these relationships are quite robust and that there was sufficient power to accurately detect the effects of interest with our sample sizes. Another potential concern was that although preparation level was manipulated by delivering the stimulus either early or late in the foreperiod, the variable foreperiod may have also contributed to variability in preparation levels due to increasing go-signal predictability as the foreperiod progressed. To examine this possibility, the effects of foreperiod duration were examined with regards to response triggering and RT latency (see “Supplementary Analysis”), which confirmed that preparation levels were not substantially affected by the variable foreperiod.

In summary, the current study provides new and important information pertaining to the conditions under which a loud auditory stimulus results in triggering of a prepared response. It is clear that the proportion of response triggering and SCM+ incidence can be increased by using a more intense (≥ 120 dB) stimulus presented when participants are at maximal preparatory activation. Although any approach to categorizing behavioural effects is unlikely to result in 100% accuracy, during StartReact protocols SCM activation appears to be a robust and reliable predictor of response triggering regardless of the acoustic stimulus parameters.

## Methods: Experiment 1

### Participants

Nineteen adults with normal or corrected-to-normal vision and no upper body abnormalities participated in this study. As the purpose of the experiment was to examine whether response triggering is dependent upon SCM incidence, those participants that did not show a reliable startle indicator (> 50%; see criterion below) for the loudest auditory stimulus (120 dB) presented 300 ms prior to the go-signal were excluded from the analysis, resulting in a final sample size of 11 participants (3M:8F; M_age_ = 27.1, SD = 7.2). Although our exclusion criterion resulted in a relatively small sample size, previous studies using a StartReact protocol have shown large effect sizes for stimulus intensity (η^2^_p_ > 0.7)^22^ and stimulus presentation time (η^2^_p_ > 0.9)^30^, indicating that the current sample size resulted in sufficient power to detect the effects of interest. All participants provided written informed consent, and the experimental procedure was approved by the University of Ottawa Research Ethics Board and were in accordance with the seventh revision of the Declaration of Helsinki.

### Apparatus and task

Participants sat facing a 24″ computer monitor (Asus VG248; 144 Hz refresh) with the right arm resting parallel to the floor in a custom manipulandum that restricted movement to flexion and extension of the wrist. The task required participants to begin from a neutral position and perform a 20° wrist extension in response to a visual go-signal. An extension movement was chosen, as opposed to a flexion movement, to ensure the prepared response could be easily differentiated from the startle reflex, which is typically expressed as a generalized flexion response^38^. Each trial began with a 6 cm black square frame (3 mm thickness) being displayed on the light grey computer screen. Following a 2500–3000 ms variable foreperiod, the square filled in bright green as the imperative “go” stimulus. On selected trials, an auditory stimulus of 80 dB, 100 dB, 110 dB, or 120 dB (25 ms, white noise waveform) was presented either 1000 ms or 300 ms prior to the visual go-signal. Participants first completed a practice/familiarization block of 10 trials with the visual stimulus only. This was followed by four testing blocks of 32 trials, with each block consisting of 16 visual only trials and two trials of each of the four auditory stimulus intensities presented at 1000 ms and 300 ms prior to the visual stimulus (i.e., 16 auditory trials per block; two trials each of four stimulus intensities at two presentation intervals). Trial presentation was pseudorandom such that neither of the two most intense auditory stimuli (110 dB/120 dB) was presented on the first two trials of a block, or on two consecutive trials. Participants were informed they may hear a sound during the trial that was irrelevant to the task, but were not corrected if they responded prior to the visual stimulus, as it was expected that early response triggering by the auditory stimulus would occur on some trials. A points system was provided to motivate participants and encourage fast RTs whereby the visual displacement RT resulted in 1 positive point for every 2 ms below 230 ms (up to a maximum of 25 points) and 1 negative point for every 2 ms above 350 ms (up to maximum of 25 points). Feedback regarding RT, movement accuracy, points per trial, and total points were provided following each trial.

### Instrumentation and stimuli

All auditory stimuli were generated using digital to analog hardware (National Instruments PCIe-6321), and amplified and presented by a loudspeaker (MG Electronics M58-H, frequency response 300 Hz–11 kHz, rise time < 1 ms) located 30 cm behind the participant at ear level. Stimulus intensity was confirmed using a precision sound level meter (Cirrus Research Optimus, CR:162C; A-weighted, impulse setting) placed at the location of the participant’s left ear during testing. Surface EMG data were collected from the muscle bellies of the right extensor carpi radialis (ECR; agonist), right flexor carpi radialis (FCR; antagonist), and right SCM (startle indicator) using bipolar preamplified surface electrodes that were connected to an external amplifier system (Delsys Bagnoli-8). Wrist angular position data were collected using a potentiometer attached to the axis of rotation of the manipulandum. Raw band-passed (20–450 Hz) EMG and potentiometer data were digitally sampled at 4000 Hz (National Instruments PCI-6024E) using a customized LabView program. Data collection began 2000 ms prior to the imperative stimulus and continued for 4000 ms.

### Data reduction and analysis

EMG burst onsets were identified for all muscles on each trial using a thresholding algorithm that identified the point at which EMG activity began a sustained increase above baseline. The temporal location of this point was determined as the first point where the DC offset corrected, rectified, and filtered (25 Hz low-pass elliptic filter) EMG signal increased more than 2 standard deviations above the level of activity observed in baseline (mean of 500 ms of activity immediately prior to the earliest possible auditory stimulus onset), and remained elevated for at least 25 ms^39^. This point was visually confirmed and corrected if misidentified due to the strictness of the algorithm (e.g., if the agonist/antagonist EMG onset was clearly incorrect based on the positional data). Auditory stimulus trials were designated as SCM+ or SCM− based on the presence (SCM+) or absence (SCM−) of a burst of SCM EMG occurring in the SCM within 20–120 ms of presentation of the auditory stimulus^21^.

The auditory stimulus trials were categorized as either auditory RT trials or visual RT trials, depending on the stimulus to which the participants responded. Those trials in which the response was initiated prior to the visual go-signal were assumed to be triggered by the auditory stimulus, whereas those trials in which the response was initiated following the visual go-signal were considered to be in response to the visual stimulus. When a response occurred to the auditory stimulus, premotor RT was defined as the time interval between the auditory stimulus and EMG onset in the ECR, whereas premotor visual RT was defined as the time interval between the visual go-signal and EMG onset in the ECR (FCR activation was not analyzed). Trials in which an error occurred were discarded, including anticipation (RT < 50 ms, 13 trials), slow RT (auditory RT > 300 ms, 14 trials; visual RT > 400 ms, 6 trials), or movement errors not captured by the other categories (6 trials). This resulted in a total inclusion rate of 97.3% (1370/1408). Practice trials were not analyzed.

### Statistical analysis

#### Startle indicators

The mean proportion of auditory trials that led to an observed burst of EMG activity in SCM (corrected for normality using an arcsine square root transformation) was analyzed using a 2 Presentation Time (early/− 1000 ms; late/− 300 ms) × 4 Stimulus Intensity (80 dB, 100 dB, 110 dB, 120 dB) repeated measures analysis of variance (ANOVA).

#### Incidence of response triggering

The mean proportion of trials triggered by the auditory stimulus (i.e., auditory RT trials; corrected for normality using an arcsine square root transformation) was analyzed using a 2 Presentation Time (early/− 1000 ms; late/− 300 ms) × 4 Stimulus Intensity (80 dB, 100 dB, 110 dB, 120 dB) repeated measures ANOVA.

#### Premotor RT

Premotor RT latency was analyzed using linear mixed effects (LME) analyses. LME models were used as they allow all data points to be retained (e.g., multiple trials from any condition for the same participant can be included in the analysis) without violating assumptions of independence^40^. For trials where a response was made to the auditory stimulus, SCM presence (SCM+, SCM−), Stimulus Intensity (80 dB, 100 dB, 110 dB, 120 dB) and Presentation Time (late/− 300 ms; early/− 1000 ms) were specified as interacting fixed factors, and intercepts for subjects were specified as a random effect, with random slopes specified for the effects of SCM presence by subject (e.g., [model = premotor RT ~ SCM * Intensity * Time + (1 + SCM|subject)]). This model was chosen as it was the most complex random slopes model that could be successfully fit to the data.

#### Predictors of response triggering

In order to determine which experimental factors and measured variables were predictive of early response triggering, data from trials where an auditory stimulus was presented were modelled using binomial generalized linear mixed model analysis. This analysis can be thought of as a repeated measures logistic regression to predict the binary outcome of whether or not a response would be triggered by the auditory stimulus. To determine which factor(s) best predicted whether a response would be triggered early, the independent variables of auditory stimulus presentation time and stimulus intensity were included as categorical predictors, and the dependent variables of SCM presence and RT were used as categorical and continuous predictors, respectively. Models were fit using individual predictors, multiple predictors including interactions between each independent variable and each dependent variable, and a full model including all four predictors. Participants were specified as a random effect to model individual variation in the response to each of the factors. The Akaike Information Criterion with correction for small sample sizes (AICc) was used to determine the model that best fit the data. More specifically, the model with the smallest AICc was chosen. For categorical predictors, estimated marginal means of the log odds of each level of the predictor were compared using a Tukey correction for multiple comparisons.

The significance value for all statistical tests was set at p < 0.05. For ANOVA results, post-hoc pairwise comparisons were performed using Tukey’s correction for multiple comparisons. For LME results, pairwise contrasts and effect size calculations were conducted using the emmeans function in R with Tukey’s correction for multiple comparisons. Random slopes were omitted for models where the resulting fit was singular or where the model did not converge. Estimated marginal means with 95% CI are reported for post-hoc tests. Data used in LME models was examined for homoscedasticity and approximate normal distribution of residuals as well as scanned for influential cases. Analyses were performed with R statistical software^41^ and used the lme4 package^42^ along with the lmerTest package^43^, the AICctab function from the bbmle package^44^, and the emmeans package^45^.

## Methods: Experiment 2

The Methods for Experiment 2 were identical to those for Experiment 1 with the following modifications. Fifteen adults with normal or corrected-to-normal vision and no upper body abnormalities participated in this study. Similar to Experiment 1, those participants that did not show a minimum 50% SCM+ trials for the loudest auditory stimulus (120 dB) were excluded from the analysis, resulting in a final sample size of 10 participants (1M:9F; M_age_ = 23.1, SD = 3.3). Two participants from Experiment 1 also completed Experiment 2.

Participants completed a practice/familiarization block of 10 visual-go trials, followed by four testing blocks of 30 trials, with each block consisting of 24 visual only trials and three trials of each of the two SAS intensities (120 dB, 114 dB), presented 300 ms prior to the visual go-signal. This distribution resulted in auditory stimuli being presented on 20% of the trials (24/120), as compared to the 50% auditory trial rate from Experiment 1 (64/128). Trial presentation was pseudorandom such that auditory stimulus trials were never presented on the first two trials of a block, or on consecutive trials.

Trials in which an error occurred, such as anticipation (RT < 50 ms, 24 trials), slow RT (visual RT > 400 ms, 35 trials), or other movement errors not captured by the other categories (6 trials) were removed from analysis. This resulted in a total inclusion rate of 95% (1135/1200). Practice trials were not analyzed.

### Statistical analysis

#### Startle indicators

The mean proportion of SAS trials that led to an observed burst of EMG activity in SCM (corrected for normality using an arcsine square root transformation) was analyzed between stimulus intensity conditions (114 dB versus 120 dB) using a paired samples Student’s t-test.

#### Incidence of response triggering

The mean proportion of trials triggered by the SAS (corrected for normality using an arcsine square root transformation) was analyzed between stimulus intensity conditions (114 dB versus 120 dB) using a paired samples Student’s t-test.

#### Premotor RT

Auditory premotor RT latency was analyzed using LME analyses with SCM presence (SCM+, SCM−) and Stimulus Intensity (114 dB, 120 dB) specified as interacting fixed effects, and intercepts for subjects were specified as a random effect.

## Supplementary Information

Supplementary Information.
