# Supplementary material for: Response triggering by an acoustic stimulus increases with stimulus intensity and is best predicted by startle reflex activation
Source: Sci Rep. 2021 Dec 8;11:23612. doi: 10.1038/s41598-021-02825-8 (PMC8655082; doi:10.1038/s41598-021-02825-8)
Supplement: Supplementary file 1 — Supplementary Information. [file 41598_2021_2825_MOESM1_ESM.docx]

**Response triggering by an acoustic stimulus increases with stimulus intensity and is best predicted by startle reflex activation**

Dana Maslovat, Christin M. Sadler, Victoria Smith, Allison Bui, and *Anthony N. Carlsen

**Supplementary Analysis**

The use of a variable foreperiod of 2500-3000ms may have allowed for increased temporal predictability of the go-signal as time progressed due to aging foreperiod effects. To quantify whether responses that were triggered by the auditory stimulus would be more likely to have longer foreperiod durations, we examined the mean foreperiod duration on trials where a response was or was not triggered by the auditory stimulus, as a function of stimulus presentation time using linear mixed effects [model = foreperiod ~ Response * Stimulus time + (1| subject)]. The result of this analysis showed that there was a main effect of Response, F(1,118.1=5.226), p=.024, whereby responses that were triggered early tended to have slightly longer foreperiod durations than those that were not triggered by the auditory stimuli (2761ms versus 2733ms). Importantly, there was no main effect of stimulus presentation time, F(1,674.7)=1.001, p=.317, and no interaction between the factors, F(1,675.0)=0.959, p=.328, indicating that this difference was common to both stimulus presentation times. The only other primary potential metric to examine how foreperiod duration impacted preparation was reaction time (RT). As such, we assessed the impact of foreperiod duration on RT in two ways. First, we examined the effect of foreperiod duration on RT for trials where no auditory stimulus occurred. RT was slightly, (but not significantly, F(1,579.1)=0.497, p=.481), faster with longer foreperiod durations: RT decreased by 10.2ms per extra second of foreperiod, resulting in RTs that were 5.1ms faster for the longest foreperiod as compared to the shortest. Second, we examined RT when responses were triggered by the auditory stimulus. Again, RT was found to be slightly (but not significantly, F(1,219.0)=0.521, p=.471) faster with longer foreperiod durations: RT decreased by 13.3ms per second of extra foreperiod duration, resulting in RTs that were 6.7ms faster for the longest foreperiod as compared to the shortest. In sum, these analyses provide confirmation that preparation level was not substantially impacted by the variable foreperiod duration in such a way that it may have influenced our reported results or conclusions.
